# Genome-wide analysis of differentially expressed profiles of mRNAs, lncRNAs and circRNAs in chickens during *Eimeria necatrix* infection

**DOI:** 10.1186/s13071-020-04047-9

**Published:** 2020-04-03

**Authors:** Xian-Cheng Fan, Ting-Li Liu, Yi Wang, Xue-Mei Wu, Yu-Xin Wang, Peng Lai, Jun-Ke Song, Guang-Hui Zhao

**Affiliations:** 1 Department of Parasitology, College of Veterinary Medicine, Northwest A&F University, Yangling, 712100 China; 2 Center of Animal Disease Prevention and Control of Huyi District, Xi’an, 710300 China; 3 State Key Laboratory of Veterinary Etiological Biology, Key Laboratory of Veterinary Parasitology of Gansu Province, Lanzhou Veterinary Research Institute, Chinese Academy of Agricultural Sciences, Lanzhou, 730000 China

**Keywords:** *Eimeria necatrix*, mRNAs, lncRNAs, circRNAs, Chicken small intestine, Expression profile

## Abstract

**Background:**

*Eimeria necatrix*, the most highly pathogenic coccidian in chicken small intestines, can cause high morbidity and mortality in susceptible birds and devastating economic losses in poultry production, but the underlying molecular mechanisms in interaction between chicken and *E*. *necatrix* are not entirely revealed. Accumulating evidence shows that the long-non-coding RNAs (lncRNAs) and circular RNAs (circRNAs) are key regulators in various infectious diseases. However, the expression profiles and roles of these two non-coding RNAs (ncRNAs) during *E*. *necatrix* infection are still unclear.

**Methods:**

The expression profiles of mRNAs, lncRNAs and circRNAs in mid-segments of chicken small intestines at 108 h post-infection (pi) with *E. necatrix* were analyzed by using the RNA-seq technique.

**Results:**

After strict filtering of raw data, we putatively identified 49,183 mRNAs, 818 lncRNAs and 4153 circRNAs. The obtained lncRNAs were classified into four types, including 228 (27.87%) intergenic, 67 (8.19%) intronic, 166 (20.29%) anti-sense and 357 (43.64%) sense-overlapping lncRNAs; of these, 571 were found to be novel. Five types were also predicted for putative circRNAs, including 180 exonic, 54 intronic, 113 antisense, 109 intergenic and 3697 sense-overlapping circRNAs. *Eimeria necatrix* infection significantly altered the expression of 1543 mRNAs (707 upregulated and 836 downregulated), 95 lncRNAs (49 upregulated and 46 downregulated) and 13 circRNAs (9 upregulated and 4 downregulated). Target predictions revealed that 38 aberrantly expressed lncRNAs would *cis*-regulate 73 mRNAs, and 1453 mRNAs could be *trans*-regulated by 87 differentially regulated lncRNAs. Additionally, 109 potential sponging miRNAs were also identified for 9 circRNAs. GO and KEGG enrichment analysis of target mRNAs for lncRNAs, and sponging miRNA targets and source genes for circRNAs identified associations of both lncRNAs and circRNAs with host immune defense and pathogenesis during *E*. *necatrix* infection.

**Conclusions:**

To the best of our knowledge, the present study provides the first genome-wide analysis of mRNAs, lncRNAs and circRNAs in chicken small intestines infected with *E*. *necatrix*. The obtained data will offer novel clues for exploring the interaction mechanisms between chickens and *Eimeria* spp.
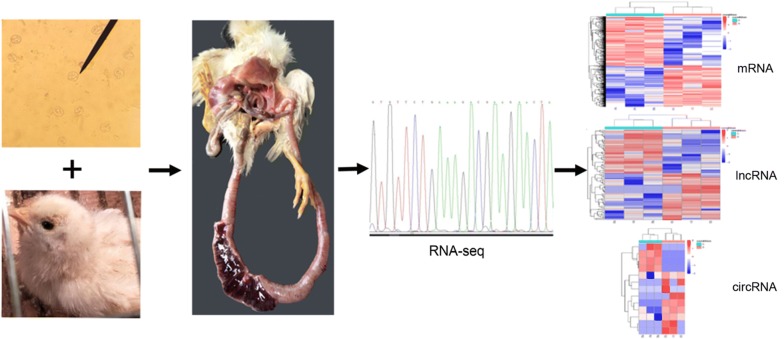

## Background

Chicken coccidiosis, caused by *Eimeria* spp., is one of the most common diseases in broilers and laying hens, seriously affecting weight gains and leading to death of birds [1, 2]. Severely infected chickens present typical clinical symptoms such as anorexia, dehydration, diarrhea with mucus and/or blood [3, 4]. The economic losses caused by chicken coccidiosis were estimated to over 3 billion USD per year worldwide and approximately 30–60 million USD have been spent to control this disease annually in China [5–7]. Currently, the prophylactic use of anticoccidial drugs or vaccines of precocious, attenuated parasites, and wild type vaccine formulations are common strategies to control chicken coccidiosis in clinic [8–11]. However, coccidiostats were challenged due to increasing evidence of drug resistance and residues [10, 12]. The vaccination was also hampered by limited production capacity, costs and the complexity of cross-protection for different *Eimeria* species [2, 13–15]. Therefore, development of novel strategies for controlling coccidiosis is urgently needed.

In recent years, finding key molecules in the host-parasite interaction is becoming an alternative approach for analyzing pathogenic mechanisms and exploring novel targets for developing drugs and vaccine candidates to control parasitic diseases [16–20]. Using the complementary DNA (cDNA) microarray technique, a great number of differentially regulated mRNA molecules in chicken intestines have been identified after infections with *E*. *tenella* [4, 21], *E*. *acervulina* [21–24] and *E*. *maxima* [9, 21, 22]. Functional analysis suggests that these differentially expressed genes would be associated with immune response/defense, apoptosis/cell death and differentiation, signal transduction, physiological development and function and extracellular matrix (ECM) [4, 9, 21–24]. However, different biological functions within the category “physiological development and function” after primary infection with these three *Eimeria* species were also predicted. For example, some differentially expressed transcripts were enriched in “connective tissue development and function” and “digestive system development and function” after *E*. *tenella* infection, while some mRNAs were enriched in “endocrine system development and function” and “hepatic system development and function” after *E*. *acervulina* infection. The biofunctions of “renal and urological system development and function” and “reproductive system development and function” were uniquely identified after *E. maxima* infection [21]. These findings suggest differences in the interaction between *Eimeria* spp. and chickens.

Recently, two novel “star non-coding RNAs (ncRNAs)”, namely long non-coding RNA (lncRNA) and circular RNA (circRNA), have been reported to play pivotal role in the interaction between hosts and bacteria [25, 26], viruses [27–30] and parasites [31–33]. For example, using the RNA-seq technique, the expression profiles of mRNAs, lncRNAs and circRNAs of tracheal tissues during *Cryptosporidium baileyi* infection were studied, and functional analysis of these differentially expressed RNAs were related to the immune system process, immune response and leukocyte activation, tight junction and glycerolipid metabolism, suggesting the important role of these RNAs in interaction between chickens and *C. baileyi* [33].

Of seven *Eimeria* species (*E. acervulina*, *E. brunetti*, *E. maxima*, *E. mitis*, *E. necatrix*, *E. praecox* and *E. tenella*) [34, 35], *E*. *necatrix* is the most highly pathogenic coccidium in small intestines of chickens, and can cause high mortality in susceptible birds [36], especially in floor-reared chickens older than 8 weeks-old [37, 38]. Currently, most studies were mainly focused on exploration of etiological biology and pathogenic features of *E*. *necatrix* [15, 39]. In the present study, the expression profiles of mRNAs, lncRNAs and circRNAs were systemically studied in chickens infected with *E*. *necatrix* using the RNA-seq technique.

## Methods

### *Eimeria necatrix* infection model

*Eimeria necatrix* used in the present study was isolated from the small intestines of sick chickens presented to the clinic (Baoji, Shaanxi, China), purified and propagated by using the single-oocyst method described previously [40, 41], and identified by using the species-specific PCR and sequencing (data not shown).The purified oocysts were passaged in 10-day-old Hy-line variety white specific-pathogen-free (SPF) chickens under a specific pathogen- and coccidia-free environment, and fresh fecal samples were collected for purifying oocysts using the salt-floatation technique. The purified oocysts were sporulated at 28 °C and oocysts with a sporulation rate > 95% were stored in potassium dichromate solution at 4 °C for further study. All sporulated oocysts were washed with sterile distilled water and treated with 5% (v/v) sodium hypochlorite solution in an ice-bath for 15–20 min before being used in infection studies [42].

One-day-old hatched Hy-line variety white cockerels were purchased from the Giant Long Company (Yangling, Shaanxi, China) and divided into the control (N) and experimental (S) groups with 30 chickens in each group. After being reared for 10 days with sterile water and fed without anti-coccidial drugs and antibiotics, in separated coccidia-free isolation cages under the specific pathogen and coccidia-free environment, each cockerel in group S was orally infected with 8000 sporulated oocysts, while the same volume of phosphate-buffered saline (PBS, pH 7.4) were orally given to cockerels in group N. Following infection, fecal samples and the mid-intestinal scrapings from the small intestines of three cockerels in both groups were examined daily for 4 days.

### Collection and preparation of tissue samples

At 108 h post-infection (pi), three cockerels of each group were randomly selected and euthanized by cervical dislocation. The mid-segment of the small intestine from each selected cockerel was excised, opened longitudinally and washed with nuclease-free PBS to remove intestinal contents and mucus. Then, the treated intestinal tissues were quick-frozen in liquid nitrogen.

### RNA extraction, library preparation and RNA sequencing

Total RNA of each frozen mid-intestinal segment was extracted using the mirVana^TM^ miRNA Isolation Kit (Ambion, Austin, TX, USA) according to the manufacturer’s instructions and stored at − 70 °C. RNA integrity was evaluated using the Agilent 2100 Bioanalyzer (Agilent, Santa Clara, CA, USA), and samples with an RNA integrity number (RIN) value of ≥ 7 were subjected to subsequent sequencing analysis. Then, the RNA library was constructed with RNA samples of 1 µg using the TruSeq Standard Total RNA with Ribo-Zero Gold in accordance with previous studies [43, 44]. RNA-seq was performed on an Illumina HisSeq^TM^ 2500 instrument generating 150 bp/125 bp paired-end reads. All these procedures were carried out in the laboratory of Shanghai OeBiotech Co. (Shanghai, China).

### Quality control and reference genome mapping

The raw data obtained were deposited into the Sequence Read Archive (SRA) within NCBI, with the accession numbers SRR9331386 and SRR9331387. The raw data were processed using the software Trimmomatic [45]. To obtain clean reads, the reads containing adaptors, low-quality reads and bases at 5ʹ- and 3ʹ-ends were removed. Meanwhile, the Q30 and GC contents of the clean reads were calculated to assess the quality of these reads. Then the clean reads were mapped into the *Gallus gallus* reference genome (ftp://ftp.ncbi.nlm.nih.gov/genomes/all/GCF/000/002/315/GCF_000002315.5_GRCg6a/GCF_000002315.5_GRCg6a_genomic.fna.gz) using hisat2 (2.2.1.0) software to obtain information of locations and sequence characteristics of the clean reads.

### Identification of lncRNAs and circRNAs from the RNA-seq dataset

The mapped clean reads were assembled based on the probabilistic model by using the software Stringtie2 (1.3.3b). Because of alternative splicing, a gene can produce isoforms with different lengths. To reduce false positives and increase the accuracy of prediction, in the present study, the known ncRNAs in the sequencing data were obtained directly, while the splicing transcripts with exons ≥ 2 and lengths > 200 bp were screened for coding potential analysis by using the software programs coding potential calculator (CPC, 0.9-r2), coding-non-coding index (CNCI, 1.0), Pfam (1.2) protein domain and predictor of long non-coding RNAs and messenger RNAs based on an improved *k*-mer scheme (PLEK1.2) [46], and the transcripts without coding potential by all four tools were predicted to be lncRNAs. The characters of identified lncRNA were then analyzed, including length distribution, GC content frequency distribution and type and number of exons.

Because chicken is not a model organism, the circRNA of the present study was *de novo* predicted using the CIRI (v2.0.3) software [47]. The clean reads were first aligned with the *G. gallus* reference genome (ftp://ftp.ncbi.nlm.nih.gov/genomes/all/GCF/000/002/315/GCF_000002315.5_GRCg6a/GCF_000002315.5_GRCg6a_genomic.fna.gz) to generate a SAM file. Then CIGAR values in the SAM file were calculated, and the paired chiastic clipping (PCC) signals in this file were also scanned. The junction reads were selected based on paired-end mapping and GT-AG splicing signals, and the clean reads including back-spliced signals and junction reads >2 were considered to be circRNAs.

### Differential expression analysis of mRNAs, lncRNAs and circRNAs

The expression abundance of mRNAs and lncRNAs was calculated using bowtie (2.2.9) and eXpress (1.5.1) programs, and the fragments per kb per million reads (FPKM) [48] were measured to determine the expression level of mRNAs and lncRNAs in each sample. At the same time, the expression of circRNAs in each sample was calculated by spliced reads per million (RPM) [49]. Then, the *DESeq* (1.18.0) package within R (3.2.0) was used to analyze the inter-sample differential expression of predicted mRNAs, lncRNAs and circRNAs. For each sample, the counts of mRNAs, lncRNAs and circRNAs were normalized to compute the fold change (FC), and the negative binomial (NB) model was used to test the significance of the differences between the groups S and N.

### Target prediction of the differentially expressed lncRNAs and circRNAs

The potential targets of the differentially expressed lncRNAs were predicted according to the co-expression correlation of lncRNAs and mRNAs by using Pearsonʼs correlation analysis (Pearson’s correlation coefficient ≥ 0.8 and *P* ≤ 0.05) calculated with Pandas DataFrame using Python (3.6.5). Two regulatory modes, namely *cis*- and *trans*-acting, were included. The differentially expressed mRNAs 100 kb upstream and downstream of the differentially expressed lncRNAs were searched for prediction of *cis* targets, while the differentially expressed coding genes not located in the same chromosome were scanned for the *trans-*regulation targets of the differentially expressed lncRNAs, with the criterion of direct complementary base pairs ≥ 10 and free energy ≤ − 50 kcal/mol.

CircRNAs have recently been identified to generally act as sponges through adsorbing miRNAs in bioprocesses of physiology and diseases [50–52], including infectious diseases. In the present study, the potential miRNA targets were predicted by using the miRanda (v3.3a) software [53], with the criterion of single-residue-pair match scores ≥ 150, ΔG ≤ − 30 kcal/mol and demand strict 5ʹ seed pairing.

### Functional analysis of mRNAs, lncRNAs and circRNAs

To predict the biological functions of mRNAs during *E*. *necatrix* infection, all differentially expressed mRNAs were submitted to the Gene Ontology (GO) (http://geneontology.org/) database to be enriched with terms of three categories, i.e. cellular component (CC), biological process (BP), and molecular function (MF), and the pathways that these mRNAs would be involved in were also predicted by conducting Kyoto Encyclopedia of Genes and Genomes (KEGG) (http://www.genome.jp/kegg/) pathway analysis. Since all differentially expressed mRNAs were predicted to be potential targets of differentially expressed lncRNAs, the functions of *cis*- and *trans*-acting targets of these lncRNAs were respectively annotated by using GO and KEGG pathway analysis. Additionally, the source genes and sponging miRNA targets of differentially expressed circRNAs were also enriched by performing GO and KEGG pathway analysis. The *P*-value was calculated within GO and KEGG pathway analysis, respectively, and a *P* < 0.05 was considered to be significantly enriched.

### Quantitative real-time polymerase chain reaction (qRT-PCR) analysis

Three mid-segments of chicken small intestines were collected from both S and N groups, and analyzed by qRT-PCR to validate the RNA-seq data. The TRIzol reagent and chloroform-isopropyl alcohol method were used to isolate the total RNA from each tissue sample for the first-strand cDNA synthesis using the PrimeScript^TM^ RT reagent Kit with gDNA Eraser (TaKaRa, Dalian, China) [54–56]. The qRT-PCR was performed using UltraSYBR Mixture (CWBIO, Beijing, China) on a Four-channel Real-time Fluorescence Quantitative PCR system (TL988; Tianlong, Shaanxi, China) using the primers listed in Additional file 1: Data S1. Three biological replicates were carried out in each qRT-PCR reaction and the *glyceraldehyde*-*3*-*phosphate dehydrogenase* (*GAPDH*) gene was included as an internal control in each reaction. The relative expression level of each gene was expressed as 2^−ΔΔCq^.

### Statistical analysis

The software Graphpad 7 (http://www.graphpad.com) was used to analyze the differences in expression levels of each selected gene between infected and control chicken samples; a *P*-value of < 0.05 was considered to indicate statistically significant differences.

## Results

### Transcriptomic sequencing data analysis

In the present study, the Illumina sequencing generated 594.8 M raw reads from six chicken intestinal samples, and the number of original sequences for each sample ranged from 98.42 M to 99.77 M. After removing adapter reads and low-quality reads, a total of 581,392,134 clean reads were retained, corresponding to 97.74% of raw sequences. The average GC content and Q30 of these clean reads were 48.35% and 95.54%, respectively. Then, these clean reads were mapped to the latest *G. gallus* reference genome for transcript assembly and annotation. The percentage of the total mapped clean reads for each sample was 93.53–96.04%, with 509,871,677 reads uniquely mapped to single loci and 41,537,429 reads mapped to multiple loci (Table 1). Then, the mapped clean reads were compared with reference transcripts by using the software cuffcompare (cufflinks-2.2.1), and 49,183 mRNAs were identified (Additional file 2: Data S2).

**Table 1:** The statistics of sequencing data

Sample ID	Raw reads	No. of clean reads	No. of mapped reads (%)	Q30 (%)	GC (%)	No. of multiple mapped (%)	No. of uniquely mapped (%)
N1	99.31 M	97,281,202	93,052,646 (95.65)	95.67	48.26	7,637,513 (7.85)	85,415,133 (87.80)
N2	99.27 M	97,187,802	91,653,917 (94.31)	95.54	48.03	6,675,495 (6.87)	84,978,422 (87.44)
N3	99.39 M	96,653,394	91,056,556 (94.21)	95.33	48.16	5,905,894 (6.11)	85,150,662 (88.10)
S1	98.42 M	96,673,328	92,845,824 (96.04)	95.93	47.85	6,629,317 (6.86)	86,216,507 (89.18)
S2	99.77 M	97,421,742	92,845,831(95.30)	95.16	48.53	6,903,683 (7.09)	85,942,148 (88.22)
S3	98.64 M	96,174,666	89,954,332(93.53)	95.61	49.26	7,785,527 (8.10)	82,168,805 (85.44)

### Identification of lncRNAs and circRNAs in chicken intestinal tissues

**Fig. 1:**
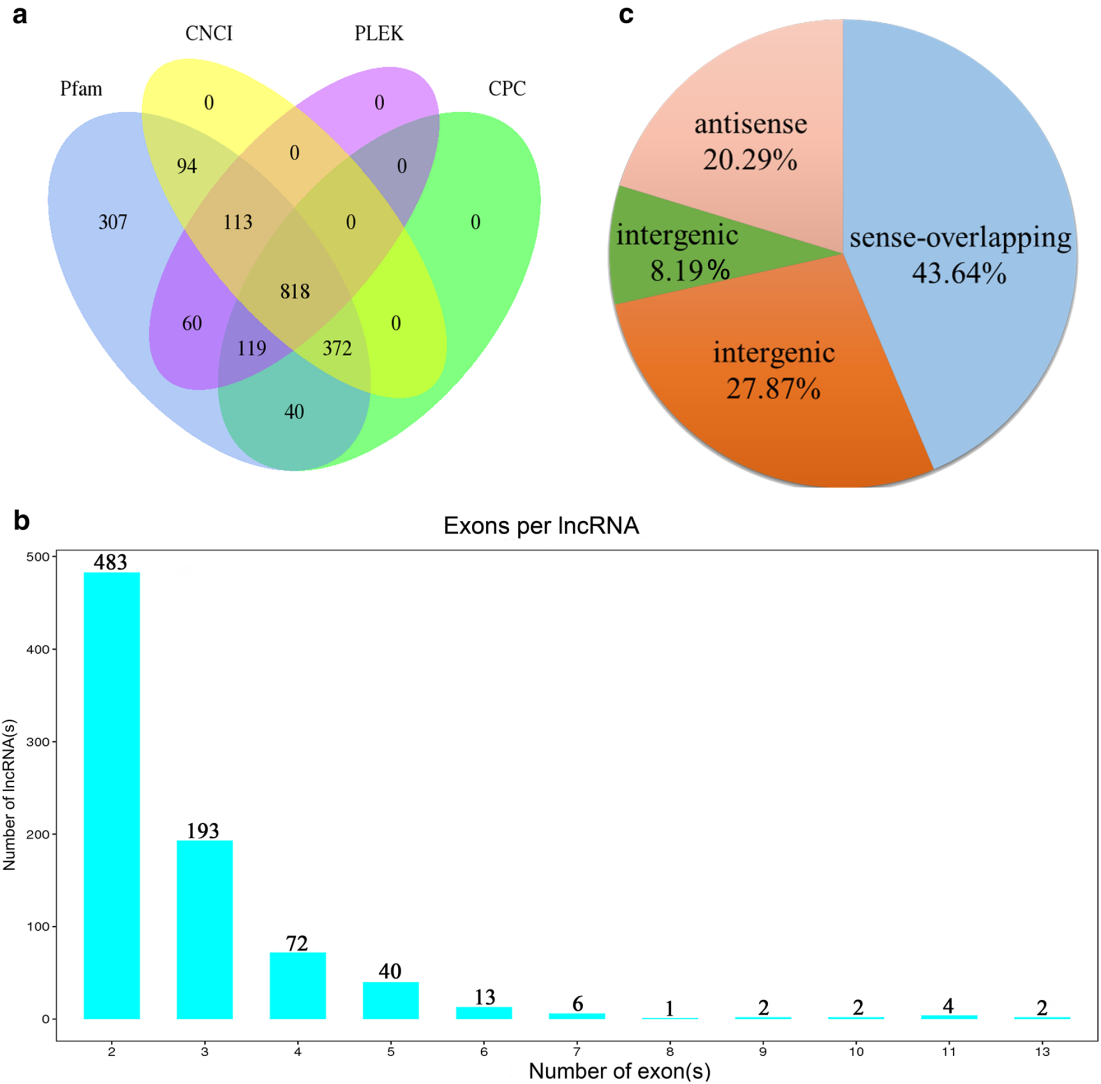
Screening, exon numbers and classification of the candidate lncRNAs in chicken small intestines. **a** Venn diagram of coding potential analysis according to strict criteria. Four tools (CPC, CNCI, Pfam and PLEK) were used to analyze the coding potential of lncRNAs. **b** The distribution of exon numbers of lncRNAs. **c** Classification of the four subtypes of lncRNAs

The mapped clean reads were assembled and compared with reference transcripts by using the software cuffcompare, and the transcripts with the characters of ‘i’ (a transfrag falling entirely within a reference intron), ‘u’ (unknown, intergenic transcript), ‘x’ (exonic overlap with reference on the opposite strand) and ‘o’ (generic exonic overlap with a reference transcript) were retained. Of them, the transcripts with the length > 200 bp and exon count ≥ 2 were selected and used for predicting the lncRNAs by screening coding potentials using the programs CPC, CNCI, Pfam and PLEK. Finally, a total of 818 lncRNAs were identified, including 247 known and 571 novel lncRNAs (Fig. 1a, Additional file 2: Data S2). The average length of these lncRNAs was 1151 bp, and most of them contained 2–5 exons (Fig. 1b, Additional file 3: Data S3). Further analysis showed that these lncRNAs were classified into four types, including 228 (27.87%) intergenic, 67 (8.19%) intronic, 166 (20.29%) anti-sense, and 357 (43.64%) sense-overlapping lncRNAs (Fig. 1c).

**Fig. 2:**
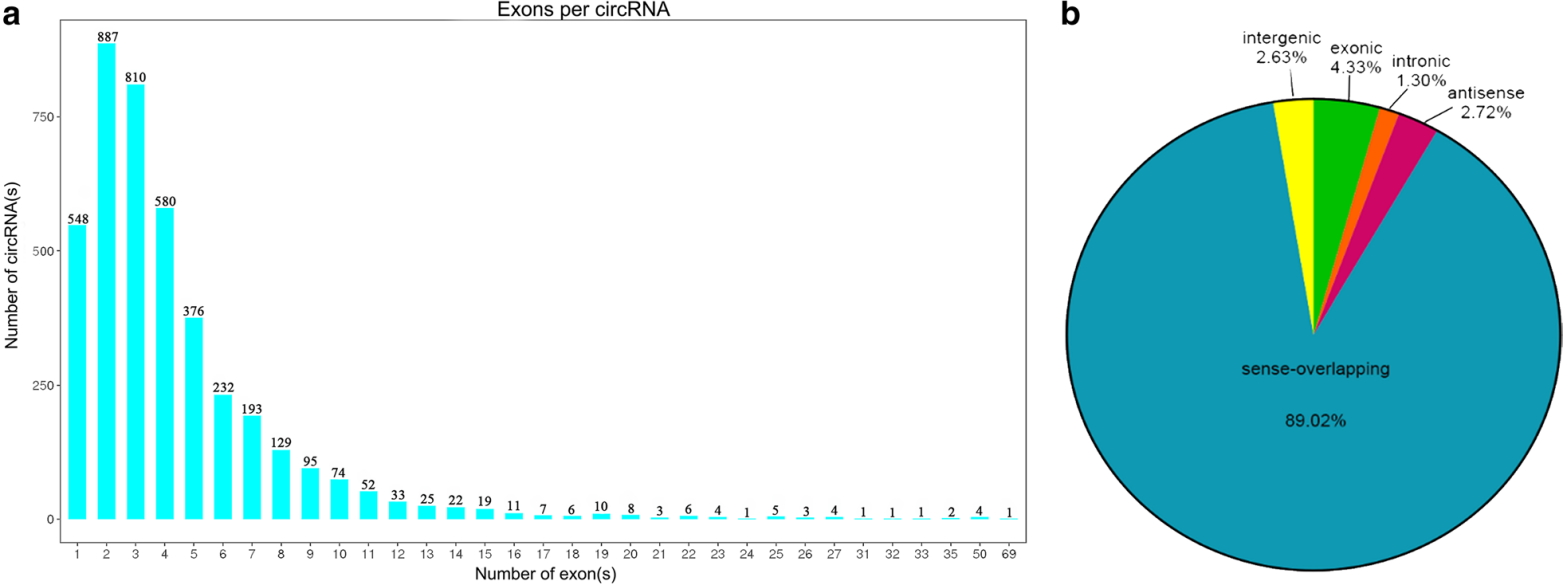
Exon numbers and classification of the candidate circRNAs in chicken small intestines. **a** The distribution of exon numbers of circRNAs. **b** Classification of the five subtypes of circRNAs

Additionally, 4153 circRNAs were predicted from the mapped clean reads in the present study (Additional file 2: Data S2). The length of these circRNAs ranged from 54 bp to 98,723 bp, with an average length of 3214 bp (Additional file 3: Data S3); the number of exons in these circRNA is shown in Fig. 2a. Of them, five types of circRNAs were predicted, including 180 exonic, 54 intronic, 113 antisense, 109 intergenic and 3697 sense-overlapping circRNAs (Fig. 2b).

### Differentially expressed profiles of mRNAs, lncRNAs and circRNAs

**Fig. 3:**
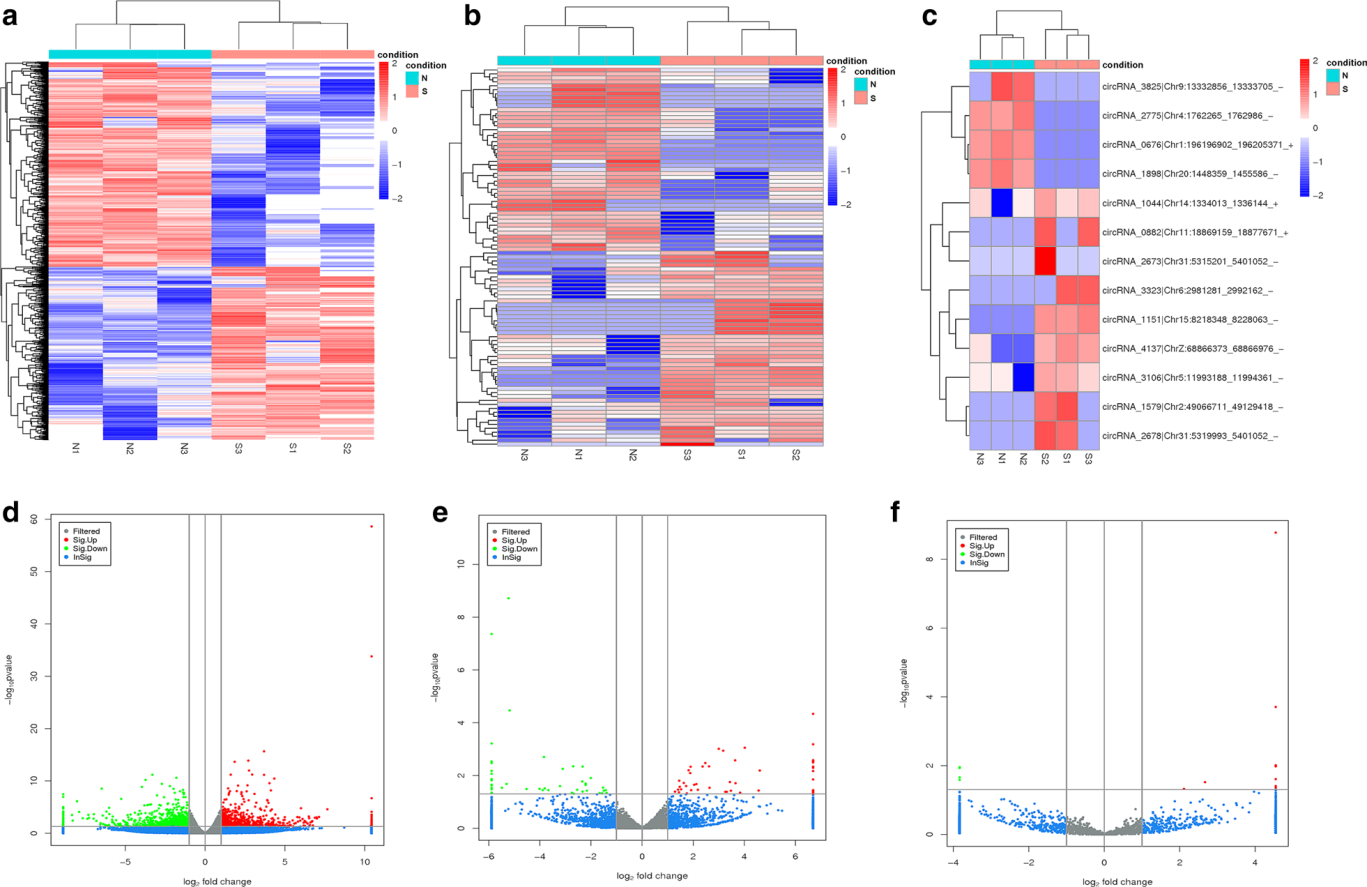
Expression patterns of differentially expressed mRNAs, lncRNAs and circRNAs in mid-segments of chicken small intestines infected with *E. necatrix* oocysts. **a** Hierarchical clustering plot showing the expression profiles of mRNAs. **b** Hierarchical clustering plot showing the expression profiles of lncRNAs. **c** Hierarchical clustering plot showing the expression profiles of circRNAs. In **a**–**c**, S1-S3 represent samples infected with *E. necatrix* oocysts and N1-N3 represent samples without infection. **d** Volcano plot showing the distributions of mRNAs. **e** Volcano plot showing the distributions of lncRNAs. **f** Volcano plot showing the distributions of circRNAs. In **d**–**f**, the significantly upregulated and downregulated mRNAs are presented as red and green dots, respectively, and the expression of mRNAs not significantly differentially expressed are presented as blue dots (FC > 2.0 and *P*-value < 0.05)

To identify the mRNAs, lncRNAs and circRNAs associated with *E. necatrix* infection in chicken small intestines, the expression of these RNAs in chickens of the groups N and S was analyzed. Hierarchical clustering of the RNAs clearly showed the different patterns of mRNAs, lncRNAs and circRNAs between the groups N and S (Fig. 3a–c). Compared with the group N, a total of 1543 mRNAs were differentially expressed (FC > 2, *P* < 0.05 and FDR < 0.05), with 707 upregulated and 836 downregulated in the S group (Fig. 3d). Additionally, 95 lncRNAs (including 9 intergenic, 4 intronic, 12 anti-sense and 12 sense-overlapping lncRNAs) and 13 circRNAs (including 10 sense-overlapping and 3 exonic circRNAs) were differentially expressed (FC > 2, *P* < 0.05 and FDR < 0.05) between the two groups. Of these, 49 lncRNAs were upregulated and 46 lncRNAs were downregulated in the S group (Fig. 3e), while 9 and 4 circRNAs were upregulated and downregulated, respectively, in this group (Fig. 3f).

### Validation of differentially regulated mRNAs, lncRNAs and circRNAs

**Fig. 4:**
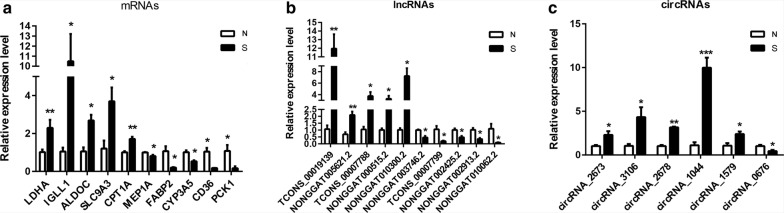
Validation of the differentially expressed genes using qRT-PCR. Three biological repeats were included for each gene. **a** The validation result for mRNAs. **b** The validation result for lncRNAs. **c** The validation result for circRNAs. *P< 0.05, **P< 0.01, ***P< 0.001

To verify the RNA-seq data, qRT-PCRs were performed to determine the expression levels of ten mRNAs (five upregulated and five downregulated), ten lncRNAs (five upregulated and five downregulated) and six circRNAs (five upregulated and one downregulated) that were randomly selected from the RNA-seq data. The expression patterns of these genes were consistent with RNA-seq (Fig. 4), confirming the high reliability and reproducibility of the RNA-seq analysis for identifying genes associated with *E. necatrix* infection.

### Co-expression analysis and target prediction

**Fig. 5:**
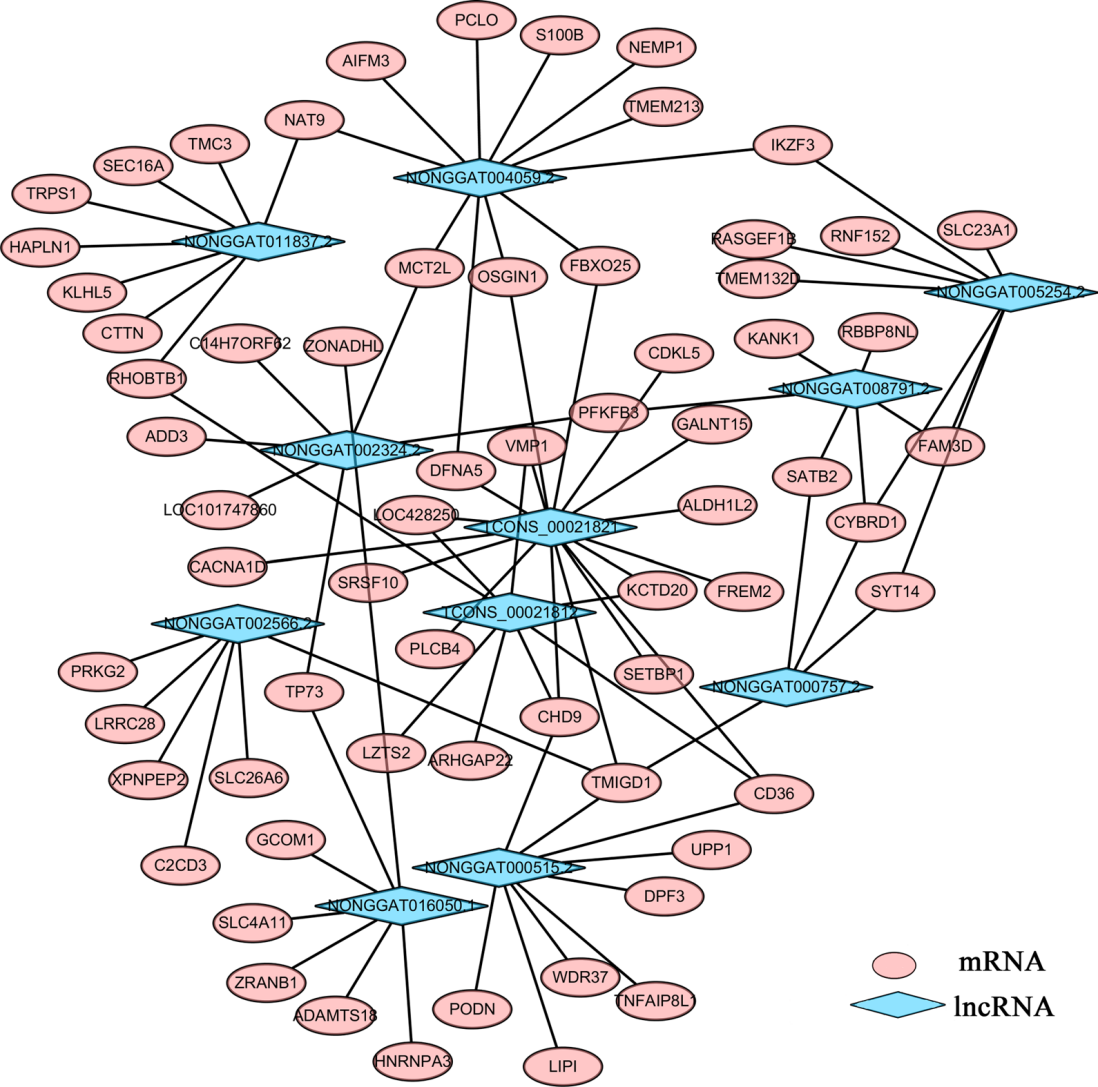
Co-repression network of significantly differentially expressed lncRNAs with their targets, with statistical relevance (Pearson’s correlation coefficient ≥ 0.8 and *P*-value ≤ 0.05) for considering co-repression

Growing evidence has indicated that lncRNA is an important competing endogenous RNA (ceRNA) in multiple physiological and disease processes through *cis*- and *trans*-regulating expression of their target mRNAs [57–59]. In the present study, the co-expression networks between the significantly differentially regulated mRNAs and lncRNAs were re-constructed. Based on expression correlation coefficients (Pearson’s correlation coefficient ≥ 0.8 and *P* ≤ 0.05), a total of 95 lncRNAs were co-expressed with 1543 mRNAs, comprising 37,134 relationships (Additional file 4: Data S4). Multiple mRNAs could be regulated by one lncRNA, and one mRNA could be regulated by several lncRNAs (Fig. 5), implying a complex regulation between differentially regulated lncRNAs and mRNAs.

To predict the potential *cis*-targets of significantly differentially expressed lncRNAs in the present study, the significantly differentially co-expressed mRNAs 100 kb upstream and downstream were searched, and 73 mRNAs would be *cis*-regulated by 38 lncRNAs (Additional file 5: Data S5). For prediction of *trans*-targets of these differentially regulated lncRNAs, the significantly differentially co-expressed mRNAs located on different chromosomes were screened, and 1453 mRNAs were identified to be potential *trans*-targets of 87 lncRNAs (Additional file 5: Data S5) with the criteria of direct complementary base pairs ≥ 10 and free energy ≤ − 50 kcal/mol.

**Fig. 6:**
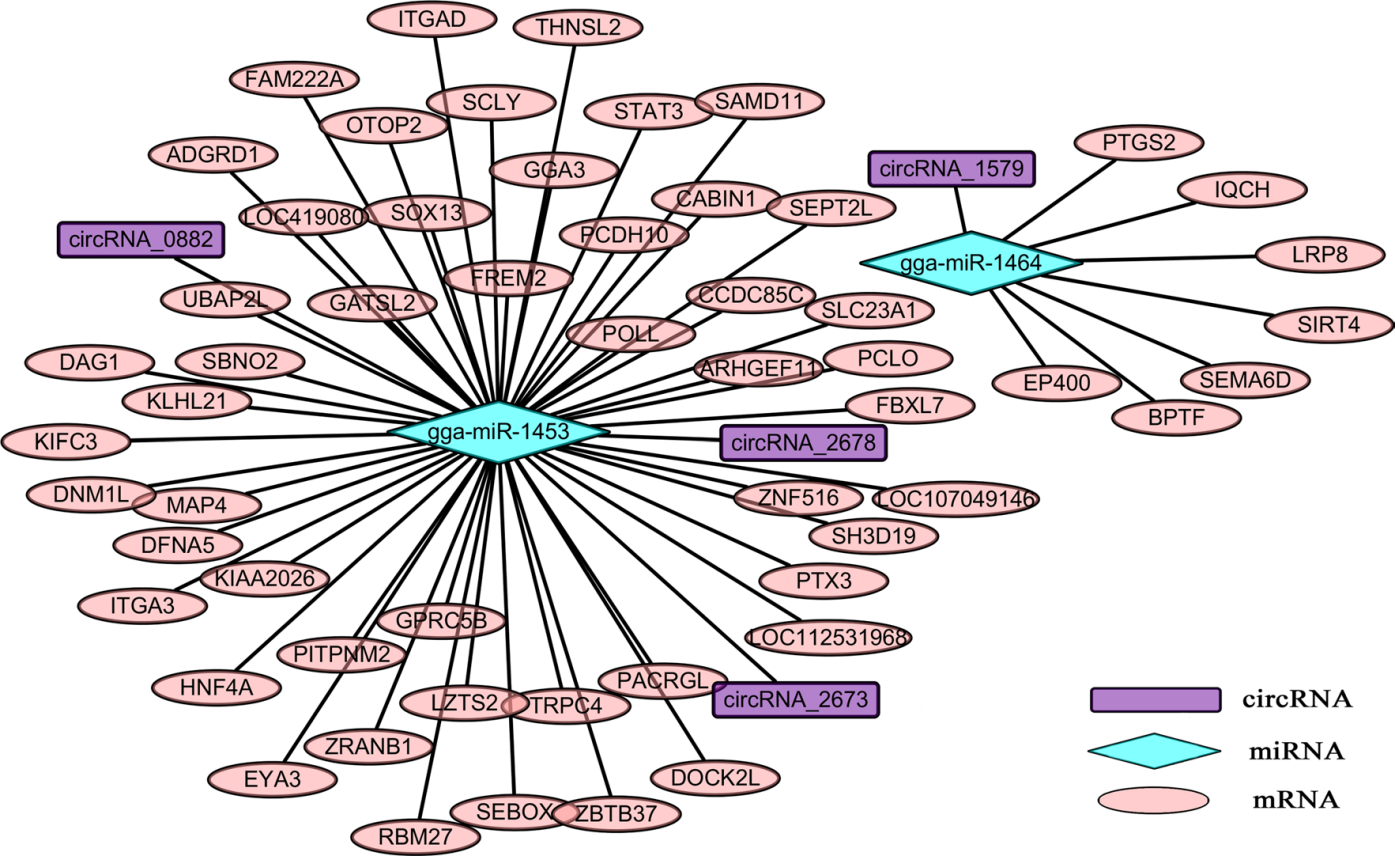
circRNA-miRNA-targets network generated using Cytoscape 3.6.1. The network consists of 4 circRNAs, 2 miRNAs and 56 targets

Recent studies evidenced that circRNAs commonly function as miRNA sponges to play significant regulatory roles in several diseases [60–64]. Herein, the potential miRNA targets of 9 significantly differentially expressed circRNAs were predicted based on complementary base pairing, with 109 miRNAs identified (Additional file 6: Data S6). The circRNA-miRNA-mRNA network showed that the regulatory relationships between circRNAs, miRNAs and target mRNAs were complex (Fig. 6).

### Functional annotation of differentially regulated lncRNAs and circRNAs

**Fig. 7:**
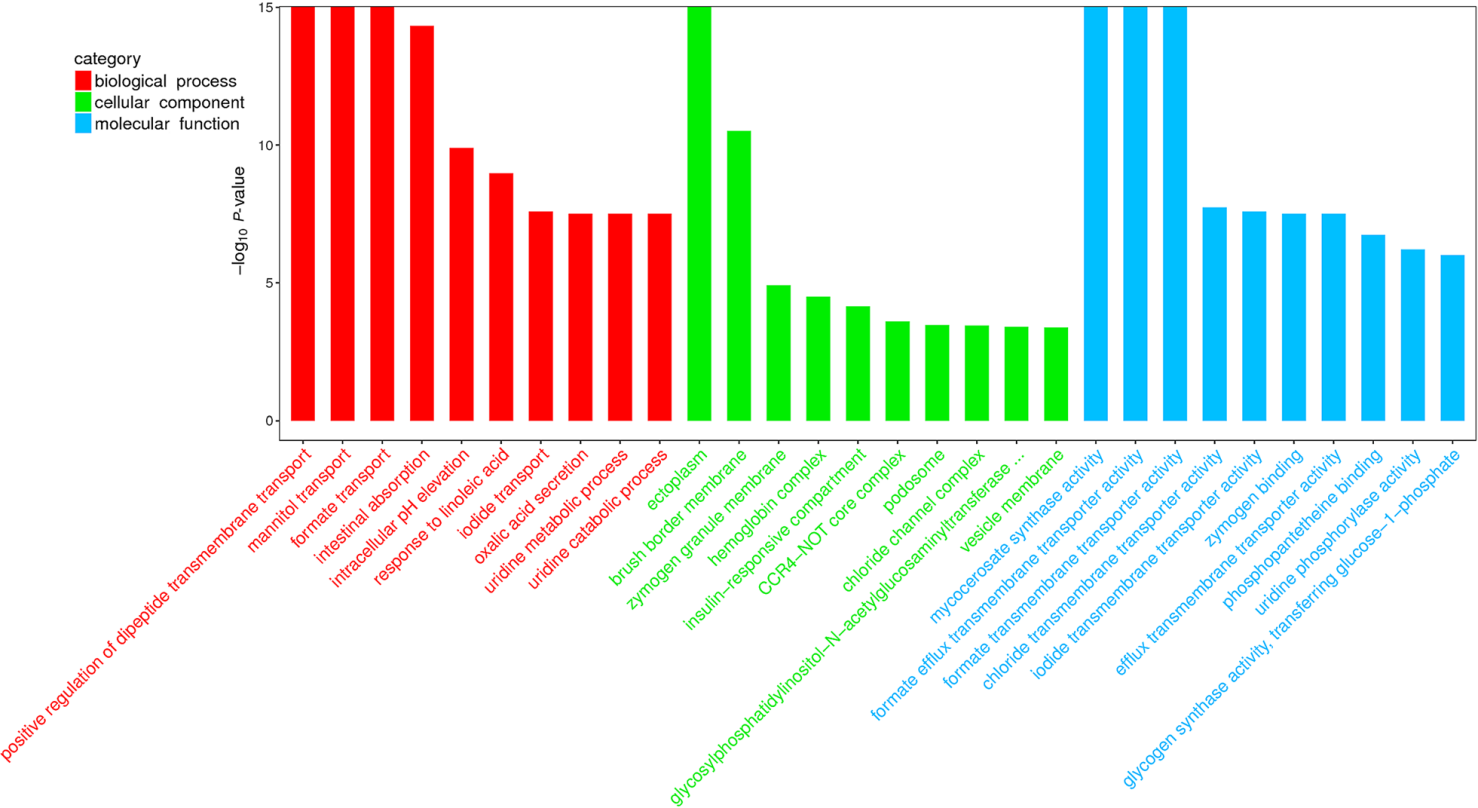
The top 10 GO terms enriched for the targets of differentially expressed lncRNAs in three categories (biological process, cellular component and molecular function), with the *P*-value ≤ 0.05 indicating significant enrichment

**Fig. 8:**
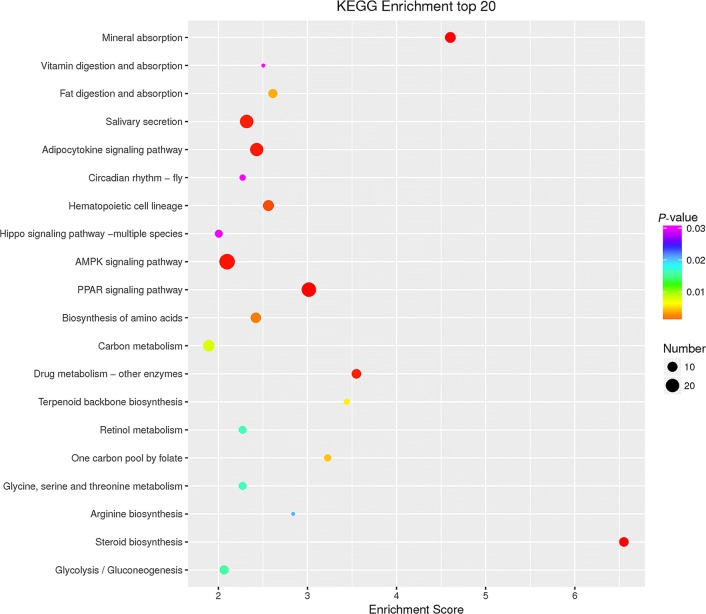
The top 20 KEGG pathway terms enriched for the targets of differentially expressed lncRNAs, with the *P*-value ≤ 0.05 indicating significant enrichment

To reveal the role of lncRNAs during *E. necatrix* infection, the potential targets of the differentially regulated lncRNA in the present study were submitted to GO and KEGG databases. A total of 1678 (*P* < 0.05) GO terms were enriched (Additional file 7: Data S7); the top 10 terms of BP, CC and MF are listed in Fig. 7. Of these, 3 (positive regulation of dipeptide transmembrane transport, mannitol transport and formate transport), 2 (ectoplasm and brush border membrane) and 3 (mycocerosate synthase activity, formate efflux transmembrane transporter activity and formate transmembrane transporter activity) terms were most significantly enriched in BP, CC and MF, respectively. The enriched GO terms of *cis*-targets included apoptotic process, cell differentiation, serine-type endopeptidase inhibitor activity, and oxidoreductase activity (Additional file 8: Data S8), while the candidate *trans*-targets were significantly enriched in the CCR4-NOT core complex, glycosylphosphatidylinositol-N-acetylglucosaminyltransferase (GPI-GnT) complex, transferring glucose-1-phosphate, cellular response to fructose stimulus, transport of mannitol, formate and iodide, metabolism of uridine, and intestinal absorption of brush border membrane (Additional file 9: Data S9). KEGG pathway analysis showed that the potential targets of the aberrantly expressed lncRNAs were enriched in 40 pathways (Fig. 8, Additional file 7: Data S7) and the most significantly enriched pathways included immunity (PPAR signaling pathway, AMPK signaling pathway and hippo signaling pathway), intestinal absorption (e.g. mineral absorption, fat digestion and absorption and vitamin digestion and absorption), metabolism (e.g. adipocytokine signaling pathway, drug metabolism, carbon metabolism, glucose, glycine, serine and threonine metabolism and retinol metabolism), biosynthesis (e.g. steroid biosynthesis, biosynthesis of amino acids and arginine biosynthesis), and hematopoietic cell lineage. The significantly enriched 31 and 40 pathways of *cis*- and *trans*-targets are listed in Additional file 8: Data S8 and Additional file 9: Data S9, respectively.

**Fig. 9:**
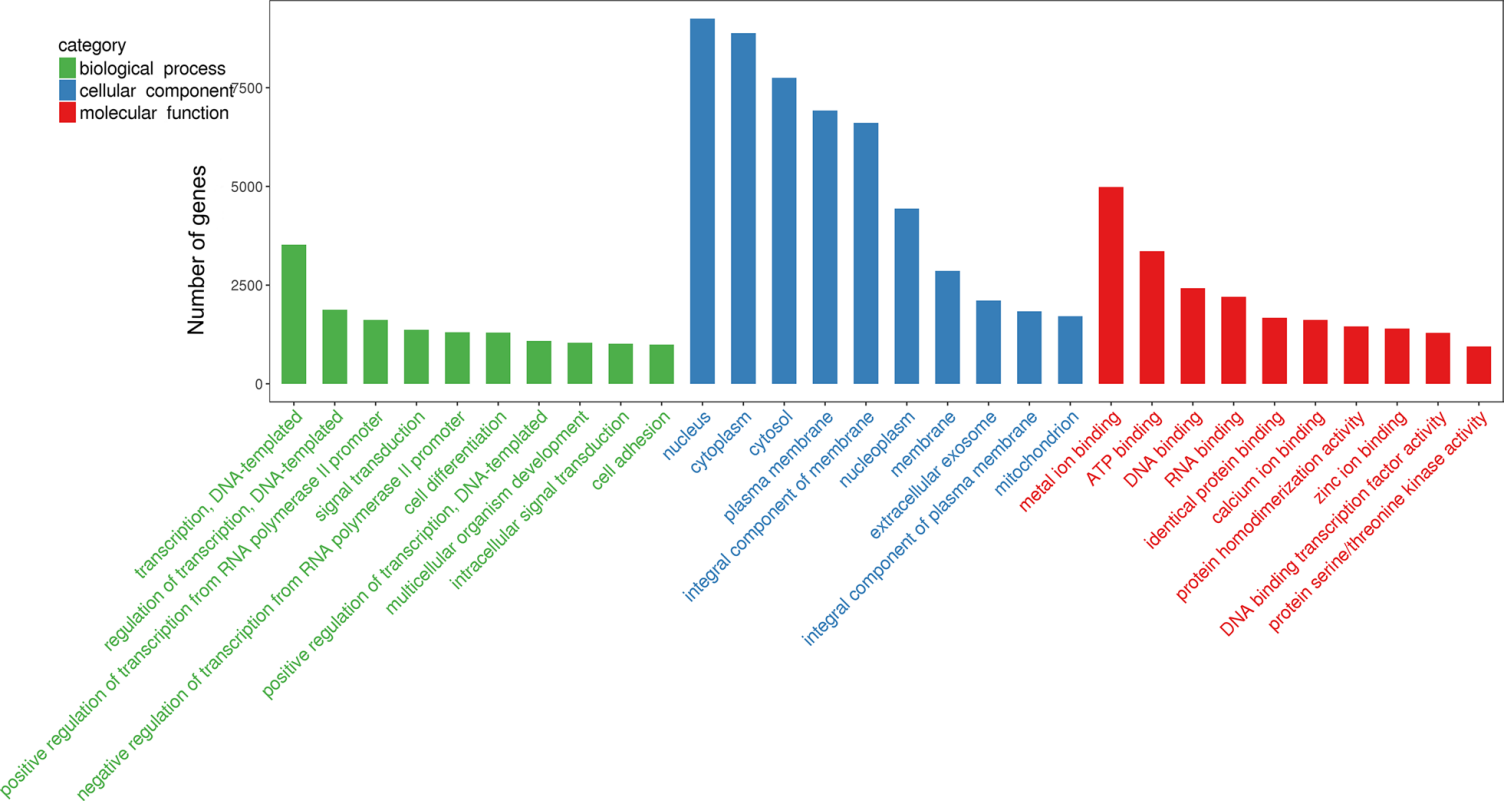
The top 10 GO terms enriched for the sponging miRNA targets of differentially expressed circRNAs in three categories (biological process, cellular component and molecular function), with the *P*-value ≤ 0.05 indicating significant enrichment

**Fig. 10:**
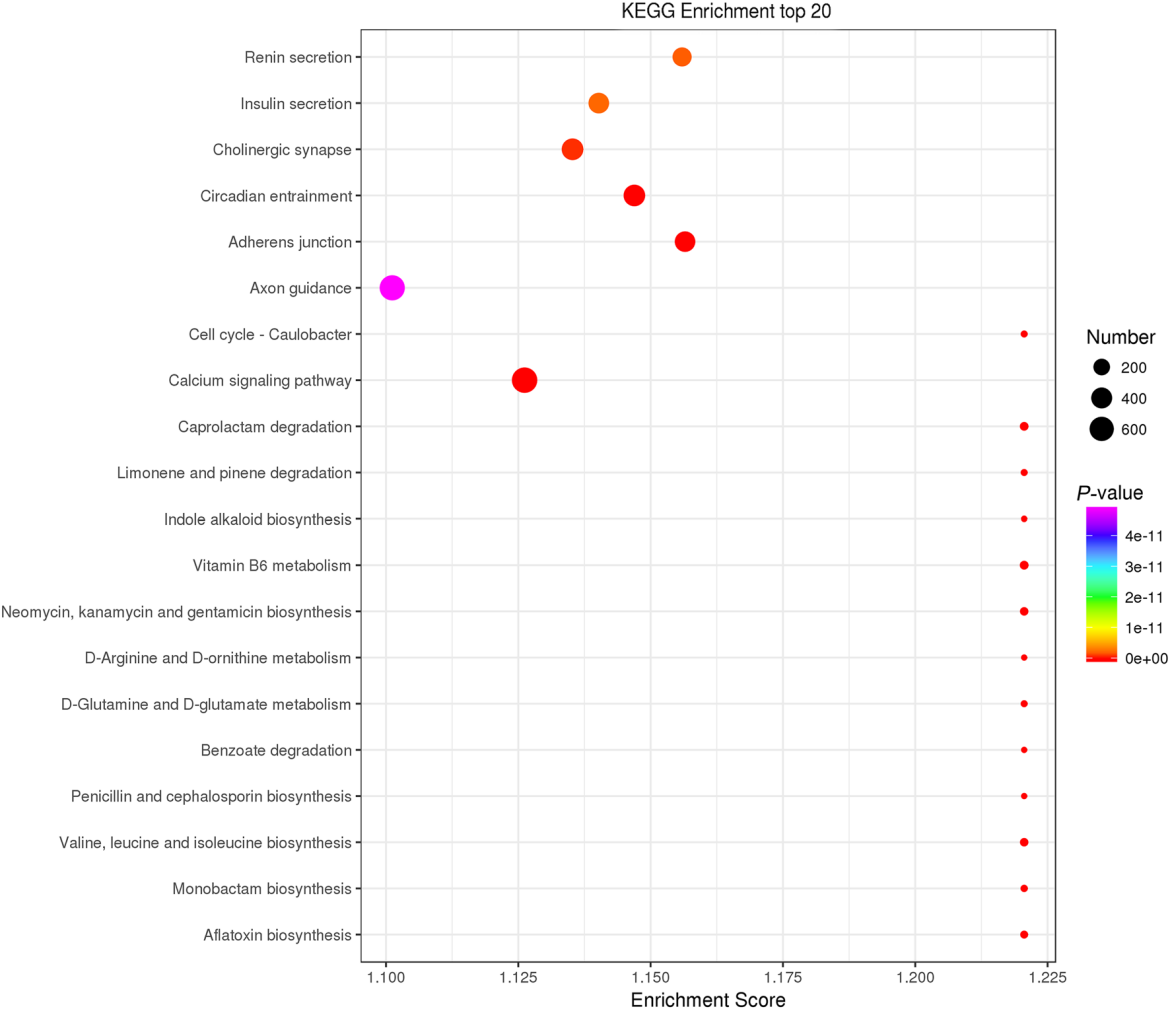
The top 20 KEGG pathway terms enriched for the sponging miRNA targets of differentially expressed circRNAs, with the *P*-value ≤ 0.05 indicating significant enrichment

The source and target genes of potential sponged miRNAs of 13 differentially expressed circRNAs were submitted to GO and KEGG pathway databases to predict the functions of these circRNAs. A total of 73 GO terms and 7 KEGG pathways were significantly enriched for source genes (Additional file 10: Data S10). Of these, 3 (Golgi membrane, Golgi apparatus, extracellular exosome) and 1 (zinc ion binding) terms were most enriched in CC and MF, respectively, while the most enriched KEGG pathways were lysosome, cell adhesion molecules (CAMs), hippo signaling pathway, inositol phosphate metabolism, phosphatidylinositol signaling system, adherens junction and rap1 signaling pathway. With regard to the sponged miRNA targets of the aberrantly expressed circRNAs, a total of 8130 GO terms and 89 KEGG pathways were significantly enriched (Figs. 9, 10, Additional file 11: Data S11).

## Discussion

In recent decades, the ncRNAs previously believed to be random transcription “noise” have been found to regulate numerous biological processes and disease development at epigenetic, transcriptional, and post-transcriptional levels [58, 59, 65]. Of these, lncRNAs were aberrantly expressed during infections of bacteria (e.g. *Helicobacter pylori*, *Talaromyces marneffei*), viruses (e.g. HIV, porcine circovirus 2, HSV-1, rabies virus, avian leukosis virus J, bovine viral diarrhea virus) and parasites (e.g. *Echinococcus granulosus*, *Cryptosporidium parvum*, *Cryptosporidium baileyi*) in animals and humans [28, 33, 66–73]. The expression profiles of host circRNAs were also elucidated after infections of avian leukosis virus J [74], bovine viral diarrhea virus [75], human cytomegalovirus [76], Orf virus [30], porcine endemic diarrhea virus [29], HIV [77], transmissible gastroenteritis virus [78], *Mycobacterium tuberculosis* [79], *Clostridium perfringens* [80] and *C. baileyi* [33]. Furthermore, the pivotal regulatory roles of some lncRNAs (e.g. PFB1055-*bsd*, SOX2OT and NR_045064) and circRNAs (e.g. hsa_circ_0001400) in these infections were well defined [81–84]. Coccidiosis, a deadly disease of chickens worldwide, seriously hampers birds’ productivity and welfare [85]. However, most previous studies mainly focused on revealing the functions of protein molecules or protein-coding genes during *Eimeria* infection [86–89]. In the present study, we first identified 818 lncRNAs and 4153 circRNAs in mid-segments of chicken small intestines at 108 h pi of *E. necatrix*, and found that 95 lncRNAs and 13 circRNAs were significantly differentially regulated due to infection. Functional analysis of these differentially expressed ncRNAs indicated that they might play significant regulatory roles in infection of *E. necatrix*.

Accumulating evidence indicates poor conservation in the nucleotide sequences of lncRNA transcripts across humans and different animal species and tissue-specificity within a single species [90, 91]. Although much efforts were made to unveil the mystery of lncRNAs in animals and humans in the last decades [58, 59, 65], the available data of lncRNAs in chicken tissues at physiological or disease conditions are limited. Using the RNA-seq technology, previous studies have identified 4404, 2481, 39,907, 8691, 1376, 6543, 2597 and 2056 lncRNAs in breast muscle, spleen, cerebrum, ovarian follicle, trachea, liver, testis and adipose of chickens, respectively [33, 92–98]. Moreover, Kern et al. [99] found 9393 lncRNAs in eight tissue types (adipose, cerebellum, cortex, hypothalamus, liver, lung, skeletal muscle and spleen) of chickens and Hong et al. [100] obtained 6900 lncRNAs genes in 20 different tissues (breast, gizzard, liver, pancreas, uterus, heart, bone marrow, kidney, cerebrum, cerebellum, eye, immature egg, mature egg, gall-bladder, comb, shank, skin, lung, spleen and fascia) of an individual Ogye, with *c.*75% of lncRNAs as tissue-specific. In the present study, 818 candidate lncRNAs were identified in the mid-segments of chicken small intestines. Of these, 571 lncRNAs, unmatched in the NCBI database and lncRNA database (http://www.noncode.org/download.php), may be considered novel, thus expanding the lncRNA database for chickens.

Previous studies have shown that lncRNAs acted as crucial regulators in the onset and progression of various infectious diseases through *cis-* and *trans*-manners [101, 102]. In the present study, *E*. *necatrix* infection significantly altered the expression profiles of 818 lncRNAs, including 247 known and 571 putative lncRNAs. However, surprisingly, all these aberrantly expressed lncRNAs were not well characterized. To better understand the potential regulatory roles of these differentially regulated lncRNAs, their *cis*- and *trans*-targets were predicted by constructing the co-expression networks of differentially expressed lncRNAs and mRNAs. A total of 73 mRNAs were potentially *cis*-regulated by 38 lncRNAs. Among them, the upregulated protein kinase C delta (PRKCD) would be regulated by the putative upregulated lncRNA NONGGAT002061.2. In the human ewing sarcoma cell line, PRKCD could activate mTOR through phosphorylation and inactivation of TSC2 [103], while the TOR pathway has been identified to be a regulator of the cell apoptosis and autophagy in chicken small intestines induced by arsenic [104] and a key player linking specific extracellular milieu and intracellular metabolism during *Salmonella* infection in chicken [105]. The downregulated ring finger protein 152 (RNF152) predicted to be regulated by the putative downregulated lncRNA NONGGAT004163.2 was localized in lysosomes and could prompt apoptosis of Hela cells [106]. In addition, 1453 mRNAs were also predicted to be *trans*-regulated by 87 lncRNAs. For example, the upregulated type I interferon receptor subunit 1 (IFNAR1) gene was regulated by 2 upregulated (TCONS_00018115 and NONGGAT001393.2) and 2 downregulated (NONGGAT002425.2 and NONGGAT011837.2) lncRNAs. Previous studies indicated that the IFNAR abundance could indirectly enhance the host defense against foreign pathogens and limit cell proliferation of hepatocellular carcinoma [107, 108]. During infection with the infectious bursal disease virus (IBDV), IFNAR1 was also upregulated in DF1 cells and the IBDV replication could be prompted through the negative regulation of CK1α on the abundance of IFNAR1 [108]. The downregulated PIK3R1 gene would be also regulated by TCONS_00018115. This gene was also downregulated during *Mycoplasma gallisepticum* (MG) infection, and it could be targeted by gga-miR-16-5p to inhibit cell proliferation and promote cell apoptosis through affecting the PI3K/Akt/NF-κB pathway, thereby exerting the anti-inflammatory effect [109]. The downregulated SAM and SH3 domain-containing protein 1 (SASH1) would be *trans*-regulated by 24 lncRNAs (including 12 upregulated and 12 downregulated) and was associated with positive regulation of the p38 MAPK cascade. MAPK has been proved to be an important pathway in the pathogenesis of apicomplexan parasites [110, 111], and the inhibition of the p38 MAPK pathway could reduce parasite motility and micronemal protein secretion and impair cell invasion of *E. tenella* [112]. Furthermore, the CD36 gene, one of significantly downregulated mRNAs in the present study, was co-expressed with 71 lncRNAs (Additional file 4: Data S4), and this gene was also downregulated during co-infections of *E. acervulina*, *E. maxima* and *E. brunetti* [113]. CD36 has been identified to be a known fatty acid transport protein to facilitate uptake of circulating non-esterified fatty acids from circulation into myocytes [114, 115]. Further analysis indicated that the CD36 could recruit the TLR4/6 receptor and induce the TLR4/6 phosphorylation after binding its ligand oxidized LDL (ox-LDL) to activate the inflammatory reaction through the NF-κB pathway [116, 117]. These findings suggest that these differentially regulated lncRNAs may have a role in immune defense against *E. necatrix* infection. On the other hand, in the present study, one upregualted mRNA (SOCS3), potentially *trans*-regulated by 32 lncRNAs (Additional file 5: Data S5), was also found to be upregulated during infection with Duck hepatitis A virus type 1 (DHAV-1). Overexpression of SOCS3 significantly inhibited the expression of IFNα, and indirectly decreased the expression of the antiviral proteins MX1 and OASL through inhibiting Janus kinase (JAK)-STAT signaling pathway, thus ultimately promoting viral proliferation and assisting in viral adaptation of DHAV-1 in chicken embryos [118]. However, SOCS3 overexpression could promote the expression of IFN-γ, and the IFN-γ has been found to play a critical role in host defense against *E*. *tenella* infection [119, 120]. Therefore, the unequivocal role of this mRNA and its regulated lncRNAs should be further determined in further studies.

To systematically and integrally understand the potential functions of lncRNAs in *E*. *necatrix* infection, we performed the GO and KEGG pathway enrichment analysis with 1543 possible target mRNAs of 95 lncRNAs. Interestingly, GO BP analysis showed that the differentially regulated mRNAs were significantly enriched in intestinal absorption, intracellular pH elevation, positive regulation of dipeptide transmembrane transport, uridine metabolic and catabolic processes, and transport of iodide, mannitol and formate. GO CC analysis revealed that these genes were clustered into ectoplasm, brush border membrane, hemoglobin complex, insulin-responsive compartment, chloride channel complex and GPI-GnT complex. GO MF analysis indicated that these targets were grouped into efflux transmembrane transporter activity, uridine phosphorylase activity, glycogen synthase activity, transferring glucose-1-phosphate, and transmembrane transporter of formate, chloride and iodide. These categories have been proven to be closely associated with pathogenic mechanisms of intestinal pathogens, including *Eimeria* spp. [4, 24, 121]. The KEGG pathway analysis demonstrated that these differentially expressed lncRNAs would be involved in immune-related biological processes (e.g. PPAR signaling pathway, AMPK signaling pathway and hippo signaling pathway), nutritional absorption (e.g. mineral, vitamin and fat), biosynthesis (e.g. steroid, terpenoid backbone and arginine) and metabolism (e.g. glucose, carbon, retinol, glycine, serine and threonine) processes, suggesting the implicated functions of these lncRNAs in the complex interplay between chicken and *E. necatrix*. Additionally, enzymes of drug metabolism were enriched by KEGG pathway analysis of both *cis*- and *trans*-targets, implicating that these genes are associated with sensitivity or resistance of drugs combating *E. necatrix*. However, these intriguing findings should be further confirmed in future research.

Of a variety of known biological potential functions (e.g. miRNA target decoys, RNA binding protein sponges and transcriptional regulators) of circRNAs [122, 123], emerging data evidenced that sponging miRNAs are now one well-recognized regulatory mechanism of circRNAs [60], although miRNA inhibition as a general feature of circRNAs is currently debated [122, 124, 125]. However, in our study, regrettably, the expression profiles of miRNAs during *E. necatrix* infection were not determined. Alternatively, the potential target miRNAs of the significantly aberrantly expressed circRNAs were just predicted based on sequence complementarity by using bioinformatics analysis, and the potential functions of these circRNAs were annotated by using GO and KEGG enrichment analysis of sponging miRNA target genes. A total of 13 significantly differentially regulated circRNAs would indirectly regulate 40,464 chicken mRNAs through sponging 109 miRNAs. GO analysis showed that these mRNAs were significantly enriched in signal transduction, regulation of DNA transcription and transcription from RNA polymerase II promoter, cell adhesion, multicellular organism development, DNA binding transcription factor activity, protein serine/threonine kinase activity, and binding of ATP, DNA, RNA, metal and calcium ions. KEGG analysis indicated that these genes would be involved in pathways of biosynthesis (e.g. valine, leucine, isoleucine, neomycin, kanamycin, gentamicin, aflatoxin and indole alkaloid), metabolism (e.g. vitamin B6, D-arginine and D-ornithine), and neurohormonal regulation (e.g. cholinergic synapse, renin secretion, insulin secretion and axon guidance). Additionally, a previous study implicated that the circRNA could compete the RNA-binding protein to affect the translation of its cognate gene [126]. Therefore, the present study also performed the functional analysis of the source genes of 13 significantly aberrantly expressed circRNAs. Of these, only 8 (5 upregulated and 3 downregulated) and 3 upregulated genes were significantly enriched in 73 GO and 7 KEGG terms, respectively (Additional file 10: Data S10). These findings suggest that circRNAs would participate in these pathways to regulate *E*. *necatrix* infection.

Notably, some limitations also exist in the present study. First, the time point for studying was limited and the dynamics of RNA expression profiles was not determined. Also, the single time point for each chicken would affect the specificity of RNA transcripts related to *E. necatrix* infection. Secondly, chicken tissues or organs other than the infection site (mid-segment of the small intestine) were not included, such as spleen and blood, in which the host immune response would be apparently reflected. Thirdly, the miRNA profiles were not examined in the present study. Since both lncRNAs and circRNAs were proved to be ceRNAs acting with miRNAs, RNA sequencing of miRNA expression would be valuable for further studying the potential regulatory roles of differentially regulated lncRNAs and circRNAs during *E*. *necatrix* infection. Last but not least, there was not enough evidence about the biological replication with only a single *E. necatrix* isolate. Although samples from three chickens for both the N and S groups were analyzed, and the purity and the oocyst sporulation rate were improved in our study, this cannot absolutely ensure that the specific RNA transcripts relate to *E. necatrix* infection. Therefore, it is of significant importance that future research investigates these limitations.

## Conclusions

The present study conducted comprehensive genomic analysis of the effect of *E*. *necatrix* infection on the expression patterns of chicken mRNAs, lncRNAs and circRNAs. The expression of 1543 mRNAs, 95 lncRNAs and 13 circRNAs was significantly altered at 108 h pi, and functional prediction using GO and KEGG pathway analysis implicated significant roles of these genes during *E*. *necatrix* infection. To the best of our knowledge, this is the first report to profile these genes in chickens during *E*. *necatrix* infection, and is also the first investigation on expression profiles of lncRNAs and circRNAs in coccidium infection. Therefore, the findings in the present study shed a new insight into understanding the underlying mechanisms of the interaction of *Eimeria* spp. and chicken intestinal cells.

## Supplementary information

**Additional file 1: Data S1.** The primers used for verification of differentially expressed mRNAs, lncRNAs and circRNAs by qRT-PCR.

**Additional file 2: Data S2.** All the expressed mRNAs, lncRNAs and circRNAs in this study.

**Additional file 3: Data S3.** The length distribution of lncRNAs and circRNAs.

**Additional file 4: Data S4.** The target genes predicted for differentially expressed lncRNAs.

**Additional file 5: Data S5.** The *cis*- and *trans*-target genes predicted for differentially expressed lncRNAs.

**Additional file 6: Data S6.** The sponging miRNAs predicted for differentially expressed circRNAs and their potential targets.

**Additional file 7: Data S7.** The GO and KEGG pathway enrichment analysis of target genes for the differentially expressed lncRNAs.

**Additional file 8: Data S8.** The GO and KEGG pathway enrichment analysis of *cis*-target genes for the differentially expressed lncRNAs.

**Additional file 9: Data S9.** The GO and KEGG pathway enrichment analysis of *trans*-targets for the differentially expressed lncRNAs.

**Additional file 10: Data S10.** The GO and KEGG pathway enrichment analysis of source genes for the differentially expressed circRNAs.

**Additional file 11: Data S11.** The GO and KEGG pathway enrichment analysis of sponging miRNA targets for the differentially expressed circRNAs.

## Data Availability

The raw data of sequencing reads are submitted to the NCBI SRA database under the accession numbers SRR9331386 and SRR9331387.

## Abbreviations

AMPK: adenosine 5ʹ-monophosphate (AMP)-activated protein kinase
BP: biological process
N: control group
CAMs: cell adhesion molecules
CC: cellular component
CCR4: CC chemokine receptor-4
cDNA: complementary DNA
ceRNA: competing endogenous RNA
circRNAs: circular RNAs
CK1α: casein kinase 1 alpha
CNCI: coding-non-coding index
CPC: coding potential calculator
DF1: Douglas Foster-1
DHAV-1: Duck hepatitis A virus type 1
ECM: extracellular matrix
FC: fold change
FPKM: fragments per kb per million reads
GAPDH: glyceraldehyde-3-phosphate dehydrogenase
GO: Gene Ontology
GPI-GnT: glycosylphosphatidylinositol-N-acetylglucosaminyltransferase
h: hour
HIV: human immunodeficiency virus
HSV-1: herpes simplex virus-1
IBDV: infectious bursal disease virus
IFNAR1: interferon receptor subunit 1
IFN-γ: interferon γ
JAK-STAT: janus kinase-signal transducer and activator of transcription
KEGG: Kyoto Encyclopedia of Genes and Genomes
lncRNAs: long-non-coding RNAs
MAPK: mitogen-activated protein kinase
MF: molecular function
MG: *Mycoplasma gallisepticum*
mTOR: mammalian target of rapamycin
NB: negative binomial
NCBI: National Center for Biotechnology Information
ncRNAs: non-coding RNAs
ox-LDL: oxidized LDL
PBS: phosphate-buffered saline
PCC: paired chiastic clipping
PH: pondus hydrogenii
pi: post-infection
PIK3R1: phosphoinositide-3-kinase regulatory subunit 1
PLEK: predictor of long non-coding RNAs and messenger RNAs based on an improved *k*-mer scheme
PPAR: peroxisome proliferators-activated receptors
PRKCD: protein kinase C delta
Q30: Qphred > 30
qRT-PCR: quantitative real-time polymerase chain reaction
RIN: RNA integrity number
RNA-seq: RNA sequencing
RNF152: ring finger protein 152
RPM: reads per million
S: experimental group
SAM: sequence alignment/map
SASH1: SAM and SH3 domain containing 1
SOCS3: suppressor of cytokine signaling 3
SOX2OT: (sex determining region Y)-box 2 overlapping transcript
SRA: sequence read archive
TLR: toll-like receptors
TSC2: tuberous sclerosis 2
